# 

**Published:** 1889-10

**Authors:** 


THE SOUTHERN SURGICAL AND GYNECOLOGICAL ASSOCIATION will meet at Nashville, Tennessee, November 12th, 13th and 14th prox. The programme for the occasion embraces thirty-eight papers to be read, (so far as reported; doubtless many more will be presented,) and the list of authors contains many distinguished names,—such as Prof. Wathen, of Louisville, one of the hardest workers and most sagacious observers in the ranks. Prof. Hadra, of Galveston; Prof. Hunter McGuire, the distinguished president; Prof. Yandell; Prof. Wm. Perrin Nicholson, of Atlanta, Ga.; Prof. Jos. Taber Johnson, of Washington, D. C., etc. This association, like "tall oaks," has, from "a little acorn" grown in a remarkably short while. Its transactions are among the most interesting and important contributions to modern medical literature.

DOCTORS, begin *now* to make preparations for the rousing meeting, the Twenty-second Annual Convention Texas State Medical Association, which will be held at Fort Worth on the fourth Tuesday in April next. Time flies and April will soon be here. Cut out your subjects, work them up; elaborate your ideas and incorporate them in a paper for some one of the sections, and be sure to write in ink, or with a type-writer. No pencil manuscript will be received, and don't forget to page your paper. This will be the grandest meeting yet, and every man will be expected to do his duty.

